# Lower-extremity amputation as a marker for renal and cardiovascular events and mortality in patients with long standing type 1 diabetes

**DOI:** 10.1186/s12933-015-0322-0

**Published:** 2016-01-07

**Authors:** Kamel Mohammedi, Louis Potier, Narimène Belhatem, Nadia Matallah, Samy Hadjadj, Ronan Roussel, Michel Marre, Gilberto Velho

**Affiliations:** INSERM, UMRS 1138, Centre de Recherche des Cordeliers, Paris, France; Department of Diabetology, Endocrinology and Nutrition, Assistance Publique Hôpitaux de Paris, Bichat Hospital, DHU FIRE, Paris, France; Univ Paris Diderot, Sorbonne Paris Cité, UFR de Médecine, Paris, France; Department of Endocrinology and Diabetology, Centre Hospitalier Universitaire de Poitiers, Poitiers, France; INSERM, Research Unit 1082, Poitiers, France; INSERM, CIC 1402, Poitiers, France; UFR de Médecine et Pharmacie, Université de Poitiers, Poitiers, France

**Keywords:** Type 1 diabetes, Amputation, Diabetic nephropathy, End stage renal disease, Myocardial infarction, Stroke, Mortality

## Abstract

**Background:**

We evaluated the risks of renal and cardiovascular complications, and mortality associated with lower extremity amputation (LEA) in patients with type 1 diabetes.

**Methods:**

We studied two cohorts of people with long standing type 1 diabetes: GENEDIAB (n = 456) and GENESIS (n = 611). Subsets of the cohorts (n = 260, n = 544) were followed for 9 and 5 years, respectively. Outcomes were the incidence of end stage renal disease (ESRD), myocardial infarction, stroke and mortality during follow-up. Analyses were performed in pooled cohorts.

**Results:**

The prevalence of LEA at baseline was 9.3 % (n = 99). A positive history of LEA was associated with the baseline prevalence of established (OR 4.50, 95 % CI 2.33–8.91, p < 0.0001) and advanced diabetic nephropathy (OR 5.50, 95 % CI 2.89–10.78, p < 0.0001), ESRD (OR 2.86, 95 % CI 1.43–5.50, p = 0.004), myocardial infarction (OR 3.25, 95 % CI 1.68–6.15, p = 0.0006) and stroke (OR 3.88, 95 % CI 1.67–8.72, p = 0.002, adjusted for sex, age, and cohort membership). A positive history of LEA at baseline was associated with the incidence during follow-up of ESRD (HR 2.69, 95 % CI 1.17–6.20, p = 0.02), and myocardial infarction (HR 3.53, 95 % CI 1.79–6.97, p = 0.0001). History of LEA was also associated with increased risk for all-cause (HR 3.55, 95 % CI 2.05–6.16, p < 0.0001), cardiovascular (HR 3.30, 95 % CI 1.36–8.02, p = 0.008), infectious disease (HR 5.18, 95 % CI 1.13–23.84, p = 0.03) and other-cause mortality (HR 2.81, 95 % CI 1.09–7.26, p = 0.03). History of LEA at baseline was associated with a 40 % reduction in the duration of survival in the subset of patients who died during follow-up. Population attributable risk of the history of LEA at baseline for total mortality during follow-up was 0.31.

**Conclusions:**

Patients with LEA have a higher risk of ESRD, myocardial infarction and cardiovascular and non-cardiovascular mortality. Our results highlight the importance of LEA as a key-predictor for major vascular events and premature death in type 1 diabetic patients.

**Electronic supplementary material:**

The online version of this article (doi:10.1186/s12933-015-0322-0) contains supplementary material, which is available to authorized users.

## Background

Lower extremity amputation (LEA) is a severe and frequent complication in patients with diabetes [1, 2]. Despite a decline of its incidence in Europe and USA in the past decades, LEA remains higher in patients with diabetes as compared to those without diabetes [3–8]. Moreover, LEA-related mortality is higher in diabetic patients than in the general population [9–11]. It is a major public health problem worldwide, with a significant social and economic burden. LEA requires lengthy hospitalizations with frequent and repeated revascularization procedures, supplemented by specialized outpatient care [12, 13]. It might result in crippling physical and psychological sequelae, with a negative impact on the quality of life, leading to social and professional disabilities [14].

Type 1 diabetes is associated with increased risk of premature death [15, 16], and this increased mortality risk is related mainly to kidney and cardiovascular complications [15, 17–19]. LEA is also associated with higher cardiovascular mortality in people with diabetes [20, 21]. However, data on LEA and risk of cardiovascular and kidney complications, and cause-specific mortality in patients with type 1 diabetes are scarce. In the present investigation we evaluated LEA-related risk of kidney and cardiovascular complications, and cardiovascular and non-cardiovascular mortality in two cohorts of patients with type 1 diabetes.

## Methods

### Participants

We studied two multicentre binational (Belgium and France) cohorts of people with long standing type 1 diabetes designed to study the vascular complications of diabetes. GENEDIAB (Génétique de la Néphropathie Diabétique) was a prospective cohort conducted, from 1994 to 2006, in 494 participants with type 1 diabetes selected on the basis of diagnosis of type 1 diabetes before the age of 35 years and past or present diagnosis of severe diabetic retinopathy [22]. The Genesis France-Belgium (GENESIS) cohort was a family-based study conducted, from 1999 to 2007, in 578 first-degree relatives and 662 probands with type 1 diabetes, selected on the basis of a diagnosis of type 1 diabetes before the age of 35 years, with initial ketosis and requirement for permanent insulin treatment within 1 year of diagnosis and past or present diagnosis of diabetic retinopathy [23]. In the present investigation, we analysed at baseline 456 GENEDIAB participants and 611 GENESIS probands for whom LEA data was available. Clinical characteristics of GENEDIAB and GENESIS participants at baseline are shown in the Additional file 1: Table S1.

### Follow-up study

In a prospective observational study, subsets of GENEDIAB (n = 260) and GENESIS (n = 544) participants were followed until an end point was reached or until February 2007. The subsets were composed of participants who attended outpatient clinics at least once during the follow-up period. Median duration of follow-up (and interquartile range) was 10.2 (2.7) and 5.1 (1.5) years for GENEDIAB and GENESIS, respectively. Clinical characteristics at baseline of GENESIS participants for whom follow-up data were available were similar to those of participants without follow-up data (Additional file 1: Table S1). GENEDIAB participants for whom follow-up data were available as compared to those without follow-up data, were older, had higher eGFR and lower prevalence of advanced diabetic nephropathy at baseline. Participants of the two cohorts gave written informed consent, and study protocols were approved by the ethics committee of Angers University Hospital (Angers, France).

### Definition of clinical parameters and outcomes

An ad hoc event committee reviewed the case record of each patient to validate the diagnosis of renal, retinal, and cardiovascular complications, peripheral sensory neuropathy, peripheral artery disease and LEA at baseline [22] and later, the incidence of outcomes during follow-up. LEA was adjudicated as amputation of a lower limb (above or below the knee, foot, toes or transmetatarsal) resulting from non-traumatic causes. Glomerular filtration rate (eGFR) was estimated by the Modification of Diet in Renal Disease (MDRD) formula [24]. Stages of diabetic nephropathy were defined as follows: no nephropathy, defined as urinary albumin concentration (UAC) <30 mg/24 h or <20 µg/min or <20 mg/l and plasma creatinine <150 µmol/l in at least 2 of 3 consecutive assessments and in the absence of antihypertensive treatment; incipient nephropathy, defined as persistent microalbuminuria (UAC = 30–300 mg/24 h or 20–200 µg/min or 20–200 mg/l) and plasma creatinine <150 µmol/l; established nephropathy, defined as past or present macroalbuminuria (UAC > 300 mg/24 h or > 200 µg/min or > 200 mg/l) and plasma creatinine <150 µmol/l; and advanced nephropathy, defined as past or present macroalbuminuria and plasma creatinine >150 µmol/l or history of end stage renal disease (ESRD), defined as haemodialysis requirement or kidney transplantation. Ocular data was obtained by direct funduscopy and/or fluorescein angiography. Diabetic retinopathy was staged according to Kohner’s classification as non-proliferative, pre-proliferative or proliferative retinopathy [25]. Myocardial infarction was diagnosed as the occurrence of at least 2 out of 3 of the following criteria: constrictive chest pain lasting 20 min or longer, increased serum creatinine phosphokinase and/or troponine levels, or typical electrocardiographic changes. Stroke was diagnosed as the occurrence of a focal neurologic deficit lasting at least 24 h, associated with evidence of brain infarction or haemorrhage by computed tomography or magnetic resonance imaging. Incidences of ESRD, myocardial infarction or stroke were defined as new cases of these outcomes during follow-up. The cause of death was established from hospital records. Missing data were completed by a phone interview with the patient’s general practitioner and/or by consulting the death certificate national registry. All-cause mortality was defined as death of any cause occurring during follow-up. Cardiovascular death was defined as death following myocardial infarction, congestive heart failure, arrhythmias and stroke. Infectious disease mortality was defined as death complicating infectious disease.

### Statistical analysis

Results are expressed as mean ± SD except when stated otherwise. Differences between groups were assessed by analysis of variance (ANOVA), analysis of covariance (ANCOVA), and Pearson’s Chi squared test. If the normality of the distribution was rejected by the Shapiro–Wilk W test, data were log-transformed for the analyses, or Wilcoxon/Kruskal–Wallis test was used. Associations of a history of LEA with the prevalence of diabetic complications at baseline were examined with logistic regression analyses. Kaplan–Meier curves were used to plot the cumulative incidences of outcomes over time by the baseline history of LEA. Cox proportional hazards survival regression analyses were used to examine the effect of explanatory variables on time-related survival (or outcome-free) rates in prospective analyses. Hazard ratios (HR) or odds ratios (OR), respectively, with their 95 % confidence intervals (CI) were computed for a positive history of LEA at baseline. Competing risk regression analysis (Fine and Gray model) was performed to estimate subhazard ratios of ESRD, myocardial infarction or stroke assuming death as a competing risk [26]. Adjustments for clinical and biological parameters were performed by including these parameters as covariates in the regression models. Cross-sectional stepwise multivariable regression analysis of covariates associated with the prevalences of the clinical outcomes at the end of follow-up were performed as sensitivity analyses. The incidence of ESRD, myocardial infarction, stroke or death during follow-up could not be ascertained in 62, 16, 30 and 7 participants, respectively. Those participants were not excluded from the study, but only from the analyses of the specific outcome where data were missing. To increase statistical power, all analyses were performed in GENEDIAB and GENESIS pooled cohorts, with appropriate adjustments for covariates to take into account cohort differences. p ≤ 0.05 was considered as significant. Statistics were performed with the JMP (SAS Institute Inc., Cary, NC, USA) and Stata (StataCorp, College Station, TX, USA) softwares.

## Results

### Clinical characteristics of participants at baseline

The prevalence of LEA at baseline was 9.3 % (n = 99) in GENEDIAB/GENESIS pooled cohorts. Characteristics of participants by a history of LEA at baseline are shown in Table 1. Individuals with a positive history of LEA as compared to other participants were more likely to be men, were older at baseline and had a longer duration of diabetes. They had higher blood pressure levels, were more likely to take antihypertensive medication and to have a history of tobacco smoking. Diabetic retinopathy, peripheral sensory neuropathy and peripheral artery disease were more frequent in individuals with LEA.

**Table 1:** Characteristics of participants by the history of lower extremity amputation at baseline

	History of LEA at baseline		p
	No	Yes	
N (%)	968 (90.7)	99 (9.3)	
Male sex (%)	51.9	70.7	0.0003
Age (years)	42.6 ± 11.6	51.6 ± 11.2	<0.0001
Age at diabetes onset (years)	15.5 ± 9.1	18.3 ± 9.0	0.007
Duration of diabetes (years)	27.0 ± 9.3	33.3 ± 9.0	<0.0001
Body mass index (kg/m^2^)	24.1 ± 3.5	23.9 ± 3.8	0.40
Systolic BP (mmHg)	134 ± 19	143 ± 20	<0.0001
Diastolic BP (mmHg)	77 ± 11	80 ± 11	0.003
HbA1c (%) and (mmol/mol)	8.5 ± 1.5 (70 ± 16)	8.8 ± 2.1 (72 ± 23)	0.31
Plasma creatinine (µmol/l)	113 ± 112	166 ± 163	<0.0001
eGFR (ml/min/1.73 m^2^)	81 ± 44	60 ± 37	<0.0001
UAC (mg/l)	22 (238)	95 (499)	0.01
Total cholesterol^a^ (mmol/l)	5.70 ± 1.45	5.67 ± 1.45	0.68
Triglycerides^a^ (mmol/l)	1.13 (0.91)	1.29 (0.5)	0.31
Tobacco smoking^b^ (%)	42.3	55.1	0.01
Antihypertensive drugs (%)	51.1	73.7	<0.0001
ACE-I or ARB drugs (%)	41.3	55.6	0.006
Lipid lowering drugs (%)	8.4	11.1	0.35
Diabetic nephropathy stages (%)	46/21/18/15	18/17/30/35	<0.0001
Diabetic retinopathy stages (%)	0/27/18/55	0/4/15/81	<0.0001
Peripheral sensory neuropathy (%)	46.4	89.7	<0.0001
Peripheral artery disease (%)	5.8	93.8	<0.0001

Results expressed as mean ± SD, except urinary albumin concentration (UAC) and triglycerides expressed as mean (interquartile range). Statistics of quantitative parameters are ANOVA performed with log-transformed data or Wilcoxon test (UAC and triglycerides). Antihypertensive drugs: all antihypertensive medication classes included. *ACE-I* angiotensin converting enzyme inhibitor, *ARB* angiotensin receptor blocker. Diabetic nephropathy stages: absence, incipient, established, and advanced nephropathy. Diabetic retinopathy stages: absent, non-proliferative, pre-proliferative, proliferative

^a^Data available only in the GENEDIAB cohort: n = 438 for total cholesterol and n = 129 for triglycerides

^b^Current or past history of tobacco smoking

p < 0.05 was significant

### LEA and kidney and cardiovascular complications at baseline

Participants with a positive history of LEA at baseline had higher UAC and lower eGFR as compared to participants without LEA history. Diabetic nephropathy at baseline was more frequent and more severe in participants with a positive history of LEA (Table 1). Regression analyses confirmed the associations of a positive history of LEA at baseline with the prevalence of established and advanced diabetic nephropathy at baseline: OR 4.50, 95 % CI 2.33–8.91, p < 0.0001 and OR 5.50, 95 % CI 2.89–10.78, p < 0.0001, respectively, in a model with minimal covariate adjustment (model 1: sex, age, and cohort membership). Associations remained significant in a multi-adjusted model taking into account differences between groups at baseline (model 2: sex, age, cohort membership, duration of diabetes, HbA1c, use of antihypertensive and lipids lowering drugs, and history of tobacco smoking): OR 4.49, 95 % CI 2.11–9.79, p < 0.0001 and OR 6.22, 95 % CI 2.92–13.73, p < 0.0001, for established and advanced diabetic nephropathy respectively. The prevalence of ESRD at baseline was 7.3 % (n = 78). It was higher in participants with a positive history of LEA than in other participants (15.2 vs 6.5 %, Pearson’s Chi squared test p = 0.002). Regression analyses confirmed the association of a positive history of LEA with the baseline prevalence of ESRD (Additional file 1: Table S2).

The prevalences of previous myocardial infarction and stroke at baseline were 5.7 % (n = 61) and 3.3 % (n = 35), respectively. Prevalences were significantly higher in participants with a positive history of LEA than in other participants: 19.2 vs. 4.4 %, respectively, for myocardial infarction (Pearson’s Chi squared test p < 0.0001), and 11.1 vs. 2.5 %, respectively, for stroke (Pearson’s Chi squared test p = 0.0001). Regression analyses, with minimal and multi-adjusted models, confirmed the association of LEA with the prevalence of previous myocardial infarction and stroke at baseline (Additional file 1: Table S2).

### LEA and clinical outcomes during follow-up

Cumulative incidences of ESRD, myocardial infarction and stroke during follow-up were 7.4 % (n = 55), 6.2 % (n = 49) and 3.4 % (n = 26), respectively. The incidence rate of these outcomes was 1.2, 1.0, and 0.5 per 100 person-years, respectively. Characteristics of participants at baseline by the incidence of each of the outcomes during follow-up are shown in the Additional file 1: Table S3. Characteristics by ESRD and myocardial infarction status during follow-up were partially published previously [27, 28]. Briefly, incident cases of ESRD, myocardial infarction or stroke during follow-up, compared to participants not presenting these outcomes, were older and had a longer duration of diabetes at baseline (except for ESRD cases), had higher blood pressure, lower eGFR (except for stroke cases), higher UAC (except for myocardial infarction cases), and were more likely to be taking antihypertensive drugs and to present peripheral sensory neuropathy and peripheral artery disease. Diabetic nephropathy and diabetic retinopathy were more frequent and more severe in incident cases of each outcome.

**Fig. 1:**
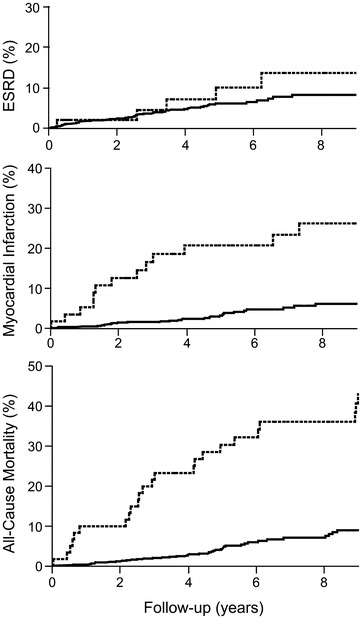
Incidence of outcomes by the history of LEA at baseline. Kaplan–Meier curves for the cumulative incidence of end stage renal disease (ESRD), myocardial infarction, and all-cause mortality during follow-up by the presence (*dashed lines*) or the absence (*solid lines*) of a history of LEA at baseline

The incidences of ESRD, myocardial infarction and stroke during follow-up were higher in participants with a positive history of LEA at baseline than in other participants (Table 2). Cox proportional hazards survival regression analyses confirmed the association of a positive history of LEA at baseline with the incidence during follow-up of ESRD and myocardial infarction both in minimal and multi-adjusted models (Table 2). No significant association was observed with the incidence of stroke. Kaplan–Meier (cumulative incidence) curves for ESRD and myocardial infarction during follow-up by the history of LEA at baseline are shown in Fig. 1. The association with the incidence of ESRD remained significant when we excluded participants with advanced nephropathy at baseline, and when we also excluded participants with established nephropathy at baseline (p = 0.003 and p = 0.03, respectively, for the multi-adjusted model; data not shown). Similarly, the association with the incidence of myocardial infarction during follow-up remained significant when we excluded from the analysis participants with a history of previous myocardial infarction at baseline, or when we included the history of previous myocardial infarction at baseline as a covariate in the model (p = 0.001 and p = 0.01, respectively, for the multi-adjusted model; data not shown).

**Table 2:** Clinical outcomes during follow-up by history of LEA at baseline

	Clinical outcomes during follow-up					
	ESRD		Myocardial infarction		Stroke	
	No	Yes	No	Yes	No	Yes
LEA at baseline						
No	644 (93.1 %)	48 (6.9 %)	697 (95.5 %)	33 (4.5 %)	698 (97.1 %)	21 (2.9 %)
Yes	44 (86.3 %)	7 (13.7 %)	43 (72.9 %)	16 (27.1 %)	51 (91.1)	5 (8.9 %)
HR (95 % CI) model 1	2.69 (1.17–6.20)		3.53 (1.79–6.97)		1.76 (0.60–5.14)	
P	0.02		0.0001		0.29	
HR (95 % CI) model 2	2.49 (1.02–6.06)		3.21 (1.50– 6.85)		1.28 (0.37–4.43)	
P	0.04		0.003		0.69	
SHR (95 % CI) model 3	2.41 (1.04–5.87)		3.16 (1.53–6.52)		1.49 (0.46–4.84)	
P	0.04		0.002		0.50	

Data expressed as number of cases and (%) by line. Hazards ratio (HR) for a positive LEA history at baseline as a risk for ESRD, myocardial infarction and stroke during follow-up, computed by Cox proportional hazards survival regressive analysis. Model 1: adjusted for cohort membership, sex and age at baseline. Model 2: adjusted for cohort membership, sex, age, duration of diabetes, HbA1c, systolic and diastolic blood pressure and diabetic retinopathy at baseline (risk for ESRD analysis), plus UAC and eGFR (risk for myocardial infarction and stroke analyses). Diabetic retinopathy was coded as an ordinal polytomic covariate: non-Proliferative (2), pre-Proliferative (3), proliferative (4). Subhazard ratio (SHR) for a positive LEA history at baseline as a risk for ESRD, myocardial infarction and stroke during follow-up, assuming death as a competing risk. SHR computed by competing risk regression analysis, adjusted for cohort membership, sex and age at baseline

p < 0.05 is significant

The associations of a positive history of LEA at baseline with the incidence of ESRD and myocardial infarction evaluated with the Cox model might be biased by the association of LEA with all-cause mortality (see below) if many patients died before achieving the renal or the coronary endpoint. Therefore, we performed competing risk regression analyses to estimate subhazard ratios (SHR) for a positive history of LEA as a risk for ESRD or myocardial infarction assuming all-cause mortality as a competing risk. SHR and HR from the Cox model were similar, indicating that death was not a competing risk in the association of LEA with ESRD or myocardial infarction (Table 2, model 3).

### LEA at baseline and mortality during follow-up

Death occurred in 72 participants during the follow-up. The cumulative incidence of all-cause mortality was 9.0 % (incidence rate 1.4 per 100 person-years). It was higher in participants with a positive history of LEA at baseline than in other participants (Table 3). Clinical characteristics of participants at baseline by the incidence of all-cause mortality during follow-up were partially published previously [27] and are shown in the Additional file 1: Table S3. Briefly, participants who died during follow-up, as compared to those who survived, were older at baseline, more frequently of the male sex, had a longer duration of diabetes, higher HbA1c, blood pressure, and UAC levels. They had lower eGFR and were more likely to have a previous history of tobacco smoking, hypertension and myocardial infarction, and to present peripheral sensory neuropathy and peripheral artery disease. Diabetic nephropathy and diabetic retinopathy were more frequent and more severe in participants who died during follow-up. Cox proportional hazards survival regression analyses showed a significant association of a positive history of LEA at baseline with all cause mortality during follow-up both in minimal and multi-adjusted models (Table 3). Kaplan–Meier (cumulative incidence) curve during follow-up by the history of LEA at baseline are shown in Fig. 1. A positive history of LEA at baseline was associated with a 40 % reduction in the duration of survival in the subset of participants who died during follow-up (2.9 ± 0.7 vs 4.8 ± 0.4 years, mean ± SD, p = 0.02, adjusted for sex, age, duration of diabetes and cohort membership). Population attributable risk of the history of LEA at baseline for total mortality during follow-up was 0.31.

**Table 3:** Mortality during follow-up by history of LEA at baseline

	All-cause mortality		Cardiovascular mortality		Infectious disease mortality		Other causes of mortality	
	No	Yes	No	Yes	No	Yes	No	Yes
LEA at baseline								
No	691 (93.6 %)	47 (6.4 %)	691 (97.5 %)	18 (2.5 %)	691 (99.0 %)	7 (1.0 %)	691 (96.9 %)	22 (3.1 %)
Yes	36 (59.0 %)	25 (41.0 %)	36 (78.3 %)	10 (21.7 %)	36 (92.3 %)	3 (7.7 %)	36 (75.0 %)	12 (25.0 %)
HR (95 % CI) model 1	3.55 (2.05–6.16)		4.04 (1.69–9.62)		3.28 (0.73 –14.68)		3.25 (1.44–7.30)	
P	<0.0001		0.002		0.12		0.004	
HR (95 % CI) model 2	2.73 (1.49–5.01)		3.30 (1.36–8.02)		5.18 (1.13–23.84)		2.81 (1.09–7.26)	
P	0.001		0.008		0.03		0.03	

Data expressed as number of cases and (%) by line. Hazards ratio (HR) for a positive LEA history at baseline as a risk for all-cause mortality, cardiovascular, infectious disease, and other causes of mortality during follow-up, computed by Cox proportional hazards survival regressive analysis. Model 1: adjusted for cohort membership, sex and age at baseline. Model 2: adjusted for cohort membership, sex, age, duration of diabetes, HbA1c, UAC, eGFR, use of antihypertensive and lipids lowering drugs, and history of tobacco smoking

p < 0.05 is significant

Causes of death during follow-up included cardiovascular complications (38.9 %), infectious diseases (13.9 %), cancer (9.7 %), acute metabolic complications (8.3 %), kidney complications (2.8 %) and other or undetermined aetiologies (26.4 %). We assessed associations of LEA with cause-specific mortality grouped as cardiovascular mortality, infectious disease mortality, and all other causes of death. For the 3 causes of mortality, the incidence during follow-up was higher in participants with a positive history of LEA at baseline than in other participants (Table 3). Cox proportional hazards survival regression analyses confirmed the association of a positive history of LEA at baseline with the incidence of cardiovascular mortality and other causes of mortality during follow-up in a model with minimal covariate adjustment (Table 3). No significant association was observed for infectious disease mortality. However, in a multi-adjusted model, associations of LEA were statistically significant with the incidence of the 3 cause-specific mortalities (Table 3). No significant association of LEA at baseline with cancer mortality was observed (data not shown).

### Sensitivity analyses

For sensitivity analyses, we performed cross-sectional stepwise multivariable regression analyses with the prevalences of diabetic nephropathy, myocardial infarction, stroke and all-cause mortality as dependent variables. To increase power, we used for each participant the most recent set of data available (follow-up, or baseline data if follow-up data was not available). Diabetic nephropathy was coded as an ordinal polytomic covariate: absence (1), incipient (2), established (3) and advanced nephropathy (4), and ESRD (5). For diabetic nephropathy analysis, sex, age, BMI, systolic and diastolic blood pressure, duration of diabetes, diabetic retinopathy stage, HbA1c, history of LEA, history of tobacco smoking, use of lipid lowering, antihypertensive and angiotensin converting enzyme inhibitor (ACE-I) or angiotensin 2 receptor blocker (ARB) drugs, and cohort membership were entered in the model as independent covariates. For myocardial infarction and stroke analyses, eGFR and UAC were also included as independent covariates. For all-cause mortality analysis, diabetic nephropathy, myocardial infarction and stroke were also included as independent covariates. Thus, use of antihypertensive and lipid lowering drugs, history of LEA, proliferative diabetic retinopathy, and systolic blood pressure remained positively associated, and age and BMI inversely associated with diabetic nephropathy (Additional file 1: Table S4), and explained ~12 % of the variation of the trait (cumulated R^2^). History of LEA, age and use of lipid lowering and antihypertensive drugs remained positively associated, and eGFR inversely associated with the prevalence of myocardial infarction (Additional file 1: Table S5), and explained ~18 % of the variation of the trait. Systolic blood pressure, duration of diabetes, UAC and history of LEA remained positively associated with the prevalence of stroke (Additional file 1: Table S6), and explained ~14 % of the variation of the trait. History of LEA, diabetic nephropathy, myocardial infarction, HbA1c, tobacco smoking, cohort membership (GENEDIAB), BMI and age remained positively associated with all-cause mortality (Additional file 1: Table S7), and explained ~36 % of the variation of the trait. History of LEA in these stepwise regression analyses explained 1.4, 9.0, 1.1 and 14.1 % of the variation of the four traits, respectively, and provided the highest single increase in cumulated R^2^ in myocardial infarction and all-cause mortality regression models.

## Discussion

In the present investigation in people with type 1 diabetes, a positive history of LEA was associated with the prevalence of established and advanced diabetic nephropathy, ESRD, myocardial infarction and stroke at baseline. LEA at baseline was also associated with increased risk during follow-up of ESRD, myocardial infarction, all-cause mortality, and specifically, with mortality due to cardiovascular and infectious diseases. When considering the participants who died during follow-up, a positive history of LEA at baseline was associated with a 40 % reduction in the duration of survival. Population attributable risk of a positive history of LEA at baseline regarding all-cause mortality during follow-up, that is, the reduction in mortality that would be observed in our cohorts in the absence of LEA was 31 %.

### Risk factors of lower-extremity amputation

As in any epidemiological study, these associations may be confounded by other factors. For instance, blood glucose control and duration of diabetes are known risk factors for LEA [20, 29, 30], but also for cardiovascular and renal complications of diabetes. However, the associations we have observed with the incidence of ESRD and myocardial infarction were independent of relevant covariates such as sex, age, duration of diabetes, blood pressure, HbA1c, UAC, eGFR, antihypertensive and lipid lowering treatments, and the current and past history of tobacco smoking, suggesting that LEA was an independent predictor for the clinical outcomes during follow-up. Moreover, despite the association of LEA with mortality, death was not a competing risk in the association of LEA with the incidence of ESRD and myocardial infarction.

### Lower-extremity amputation and risks of kidney and cardiovascular complications and mortality

Increased mortality risk associated with LEA has been reported previously in people with diabetes [10, 21], but to the best of our knowledge, this is the first study that investigates the risk associated with a history of LEA on the prevalence and the incidence of cardiovascular and kidney diseases, and specific causes of mortality in prospective cohorts of type 1 diabetes. Increased risk of mortality in type 1 diabetic individuals with a baseline history of LEA as compared to those without LEA was observed in the WHO Multinational Study of Vascular Disease in Diabetes [20]. The Danish Amputation Register, including 1406 type 1 diabetic patients, reported higher rate of mortality in patients with LEA as compared to those without, particularly during the first year following the amputation [31]. A history of LEA was also reported as an independent predictor for 5-year mortality in 1444 patients with type 1 diabetes from the Early Treatment Diabetic Retinopathy Study [32]. On the other hand, the presence of chronic kidney disease (GFR < 60 ml/min) and ESRD were associated with increased rates of mortality (46 and 290 %, respectively) in patients with diabetes who undergone LEA [33]. LEA was reported in 1.5–2.5 % of participants in Swedish, German and American surveys of people with type 1 diabetes [6, 34–36]. The higher prevalence of LEA in our cohorts at baseline (9.3 %) probably reflects the inclusion criteria, which included the presence of retinopathy for GENESIS and severe (pre-proliferative or proliferative) retinopathy for GENEDIAB. Participants from our cohorts were older, had a longer duration of diabetes and were more severely ill than participants from the other surveys.

### Strengths and limitations

LEA is the consequence of acute and chronic podiatric complications, including diabetic neuropathy, peripheral artery disease, skin microangiopathy with impairment of skin blood flow, coagulation disorders and infection complications [37–40]. The main limitation of our study was that data on specific causes of LEA and on the level of amputation (above or below the knee, foot, or toes), obtained retrospectively from hospital records, were fragmentary and could not be used in the investigation. Another limitation was the relatively small number of some of the outcomes, potentially reducing the statistical power to observe associations such as in the case of the incidence of stroke or some specific causes of death, including kidney disease-related mortality. The main strength of our study is the cross-sectional analysis of 1000 patients with long standing type 1 diabetes from two independent cohorts, with a follow-up of 5–9 years of more than 800 participants, with detailed renal, cardiovascular and survival outcomes including cause-specific mortality data. It is noteworthy that in cross-sectional analyses of total prevalence of diabetic nephropathy, myocardial infarction, stroke and all-cause mortality, LEA remained significantly associated with the all traits, including stroke.

## Conclusions

LEA reflects widespread micro- and macrovascular damage in people with type 1 diabetes. Patients with LEA have a higher risk of ESRD, myocardial infarction and premature death from cardiovascular and non-cardiovascular causes. Our results confirm the burden of LEA in patients with type 1 diabetes beyond podiatric issues, and highlight the importance of LEA as a key-predictor of severe outcomes.

## Additional file

10.1186/s12933-015-0322-0 Supplementary Tables.

## Abbreviations

ACE-I: angiotensin converting enzyme inhibitor
ARB: angiotensin receptor blocker
eGFR: estimated glomerular filtration rate
ESRD: end-stage renal disease
GENEDIAB: “Génétique de la Néphropathie Diabétique” Study
GENESIS: genesis France-Belgium cohort
LEA: lower extremity amputation
MDRD: modification of diet in renal disease formula
SHR: subhazard ratio
UAC: urinary albumin concentration
