# Supplementary material for: Lower-extremity amputation as a marker for renal and cardiovascular events and mortality in patients with long standing type 1 diabetes
Source: Cardiovasc Diabetol. 2016 Jan 7;15:5. doi: 10.1186/s12933-015-0322-0 (PMC4705699; doi:10.1186/s12933-015-0322-0)
Supplement: Supplementary file 1 — 10.1186/s12933-015-0322-0 Supplementary Tables. [file 12933_2015_322_MOESM1_ESM.docx]

**Additional file 1 - Table S1. Clinical characteristics of GENEDIAB and GENESIS participants at baseline by follow-up inclusion**

|  | GENEDIAB | | | |  | GENESIS | | | |
| --- | --- | --- | --- | --- | --- | --- | --- | --- | --- |
|  | All participants | Follow-up data: No | Follow-up data: Yes | p |  | All participants | Follow-up data: No | Follow-up data: Yes | p |
| N | 456 | 196 | 160 |  |  | 611 | 67 | 544 |  |
| Duration of follow-up (years) | - | - | 10.2 (2.7) |  |  | - | - | 5.1 (1.5) |  |
| Age (years) | 44.8 ± 12.5 | 43.5 ± 12.7 | 45.8 ± 12.2 | 0.03 |  | 42.4 ± 11.2 | 42.5 ± 11.6 | 42.4 ± 11.2 | 0.96 |
| Sex: male (%) | 55.9 | 51.0 | 59.6 | 0.07 |  | 51.9 | 44.8 | 52.8 | 0.22 |
| Age at diabetes onset (years) | 15.9 ± 8.9 | 15.1 ± 8.9 | 16.5 ± 8.8 | 0.04 |  | 15.7 ± 9.3 | 16.4 ± 10.4 | 15.6 ± 9.2 | 0.74 |
| Duration of diabetes (years) | 28.8 ± 9.8 | 28.4 ± 9.8 | 29.1 ± 9.7 | 0.47 |  | 26.7 ± 9.2 | 25.9 ± 9.4 | 26.8 ± 9.1 | 0.41 |
| Body mass index (kg/m^2^) | 23.7 ± 3.3 | 23.2 ± 3.2 | 24.1 ± 3.3 | 0.007 |  | 24.4 ± 3.6 | 24.0 ± 3.5 | 24.5 ± 3.6 | 0.35 |
| Systolic blood pressure (mmHg) | 138 ± 19 | 137 ± 20 | 139 ± 18 | 0.11 |  | 132 ± 19 | 132 ± 20 | 132 ± 19 | 0.99 |
| Diastolic blood pressure (mmHg) | 79 ± 12 | 79 ± 12 | 80 ± 11 | 0.57 |  | 75 ± 10 | 75 ± 11 | 75 ± 10 | 0.83 |
| HbA_1c_ (%) | 8.6 ± 1.8 | 8.5 ± 1.9 | 8.7 ± 1.7 | 0.14 |  | 8.5 ± 1.4 | 8.6 ± 1.3 | 8.5 ± 1.3 | 0.77 |
| HbA_1c_ (mmol/mol) | 70 ± 20 | 69 ± 21 | 71 ± 19 | - |  | 69 ± 15 | 70 ± 14 | 69 ± 15 | - |
| Plasma creatinine (µmol/l) | 126 ± 116 | 138 ± 130 | 117 ± 104 | 0.02 |  | 112 ± 120 | 114 ± 113 | 112 ± 121 | 0.43 |
| eGFR (ml/min) | 70 ± 31 | 66 ± 31 | 73 ± 29 | 0.01 |  | 86 ± 50 | 80 ± 43 | 90 ± 53 | 0.29 |
| UAC (mg/l) | 32 (428) | 35 (495) | 30 (350) | 0.20 |  | 21 (186) | 55 (431) | 19 (168) | 0.08 |
| Total cholesterol^*^ (mmol/l) | 5.69 ± 1.45 | 5.71 ± 1.43 | 5.68 ± 1.47 | 0.75 |  | - | - | - | - |
| Triglycerides^*^ (mmol/l) | 1.14 (0.82) | 1.19 (0.77) | 1.13 (0.80) | 0.37 |  | - | - | - | - |
| Tobacco smoking^†^ (%) | 47.4 | 52.3 | 43.7 | 0.07 |  | 40.6 | 34.3 | 41.4 | 0.27 |
| Antihypertensive drugs (%) | 56.1 | 54.1 | 57.5 | 0.47 |  | 51.1 | 55.2 | 50.6 | 0.47 |
| ACE-I or ARB drugs (%) | 43.7 | 41.1 | 45.6 | 0.36 |  | 41.9 | 47.8 | 41.2 | 0.30 |
| Lipid lowering drugs (%) | 8.3 | 7.7 | 8.9 | 0.65 |  | 8.8 | 10.5 | 8.6 | 0.62 |
| Diabetic nephropathy stages (%) | 33/21/24/22 | 27/21/22/30 | 37/22/25/16 | 0.002 |  | 50/21/16/13 | 37/23/25/15 | 52/20/15/13 | 0.06 |
| Diabetic retinopathy stages (%) | 0/0/20/80 | 0/0/19/81 | 0/0/21/79 | 0.72 |  | 0/43/16/41 | 0/33/15/52 | 0/44/16/40 | 0.12 |
| Peripheral sensory neuropathy (%) | 66.6 | 60.4 | 71.4 | 0.02 |  | 37.8 | 43.1 | 37.3 | 0.39 |
| Peripheral artery disease (%) | 17.9 | 15.5 | 19.7 | 0.26 |  | 11.6 | 15.3 | 11.1 | 0.35 |
| Previous Myocardial Infarction (%) | 7.2 | 8.2 | 6.6 | 0.53 |  | 4.6 | 6.3 | 4.5 | 0.54 |
| Previous Stroke (%) | 4.4 | 4.6 | 3.9 | 0.71 |  | 2.6 | 3.0 | 2.6 | 0.85 |

Results expressed as mean ± SD, except urinary albumin concentration (UAC) and triglycerides expressed as median and interquartile range. Statistics of quantitative parameters are ANOVA performed with log-transformed data or Wilcoxon test (UAC and triglycerides). eGFR: estimated glomerular filtration. ^*^Data available only in the GENEDIAB cohort: n=438 for total cholesterol and n=129 for triglycerides. ^†^Current or past history of tobacco smoking. Antihypertensive drugs: all antihypertensive medication classes included. ACE-I: Angiotensin Converting Enzyme Inhibitor. ARB: Angiotensin Receptor Blocker. Diabetic nephropathy stages: absence, incipient, established, and advanced nephropathy. Diabetic retinopathy stages: absence, non-proliferative, pre-proliferative, proliferative retinopathy. p<0.05 was significant.

**Additional file 1 - Table S2. Baseline prevalence of ESRD, myocardial infarction, and stroke by the history of LEA**

|  |  | Prevalence at baseline | | | | | | | |
| --- | --- | --- | --- | --- | --- | --- | --- | --- | --- |
|  |  | ESRD | |  | Myocardial Infarction | |  | Stroke | |
|  |  | No | Yes |  | No | Yes |  | No | Yes |
| LEA at baseline: No |  | 902 (93.5%) | 63 (6.5%) |  | 906 (95.6%) | 42 (4.4%) |  | 937 (97.5%) | 24 (2.5%) |
| Yes |  | 84 (84.9%) | 15 (15.2%) |  | 80 (80.8%) | 19 (19.2%) |  | 88 (88.9%) | 11 (11.1%) |
| OR (95% CI) model 1 |  | 2.86 (1.43 – 5.50) | |  | 3.25 (1.68 – 6.15) | |  | 3.88 (1.67 – 8.72) | |
| P |  | 0.004 | |  | 0.0006 | |  | 0.002 | |
| OR (95% CI) model 2 |  | 3.02 (1.37 – 6.38) | |  | 2.77 (1.40– 5.41) | |  | 3.49 (1.49 – 7.92) | |
| P |  | 0.007 | |  | 0.004 | |  | 0.005 | |

Data expressed as number of cases and (%) by line. Odds ratio (OR) for the prevalence of ESRD, myocardial infarction, and stroke at baseline by the history of LEA, computed by logistic regression analyses. Model 1: adjusted for cohort membership, sex and age at baseline. Model 2: adjusted for cohort membership, sex, age, duration of diabetes, HbA1c, use of antihypertensive and lipids lowering drugs, and history of tobacco smoking. p<0.05 was significant.

**Additional file 1 - Table S3. Characteristics of participants at baseline by clinical outcomes during follow-up**

|  | ESRD | |  | Myocardial infarction | |  | Stroke | |  | All-cause mortality | |  |
| --- | --- | --- | --- | --- | --- | --- | --- | --- | --- | --- | --- | --- |
|  | No | Yes | p | No | Yes | p | No | Yes | p | No | Yes | p |
| N (%) | 688 (92.6) | 55 (7.4) |  | 740 (93.8) | 49 (6.2) |  | 749 (96.7) | 26 (3.4) |  | 727 (91.0) | 72 (9.0) |  |
| Sex: M/F (%) | 54.7 | 56.4 | 0.81 | 53.8 | 69.4 | 0.03 | 54.5 | 69.2 | 0.14 | 53.5 | 70.8 | 0.005 |
| Age (y) | 44.0±11.7 | 39.3±11.3 | 0.002 | 42.8±11.2 | 52.8±13.1 | <0.0001 | 43.1±11.6 | 51.0±8.5 | 0.0005 | 42.9±11.4 | 49.5±12.6 | <0.0001 |
| Age at diabetes onset (y) | 16.2±9.2 | 14.3±8.6 | 0.08 | 15.6±9.1 | 19.1±8.7 | 0.01 | 15.7±9.1 | 17.9±8.4 | 0.15 | 15.7±9.1 | 17.7±9.3 | 0.16 |
| Duration of diabetes (y) | 27.7±9.5 | 25.2±9.7 | 0.05 | 27.1±9.1 | 33.7±11.7 | 0.0002 | 27.3±9.4 | 33.3±7.7 | 0.002 | 27.1±9.2 | 31.7±10.5 | 0.001 |
| BMI (kg/m^2^) | 24.5±3.5 | 23.3±4.0 | 0.005 | 24.3±3.5 | 24.9±3.8 | 0.21 | 24.3±3.5 | 24.2±3.8 | 0.85 | 24.4±3.5 | 23.6±3.8 | 0.05 |
| Systolic BP (mmHg) | 132±18 | 151±19 | <0.0001 | 133±19 | 145±21 | 0.0002 | 134±19 | 145±17 | 0.004 | 134±19 | 141±19 | 0.001 |
| Diastolic BP (mmHg) | 75±10 | 88±11 | <0.0001 | 77±11 | 80±11 | 0.06 | 77±11 | 79±8 | 0.20 | 76±11 | 80±11 | 0.01 |
| HbA1c (%)  HbA1c (mmol/l) | 8.5±1.4  70±15 | 9.1±1.9  76±21 | 0.009  0.009 | 8.5±1.5  70±16 | 8.9±1.5  74±16 | 0.07  0.07 | 8.5±1.5  70±16 | 8.9±2.2  74±24 | 0.45  0.45 | 8.4±1.3  69±15 | 9.4±2.3  79±25 | <0.0001  <0.0001 |
| Plasma creatinine (μmol/l) | 87±37 | 212±137 | <0.0001 | 110±110 | 141±157 | 0.02 | 111±113 | 118±71 | 0.11 | 108±108 | 169±168 | <0.0001 |
| eGFR (ml/min.1.73 m^2^) | 89±43 | 46±50 | <0.0001 | 86±47 | 70±31 | 0.01 | 83±46 | 64±22 | 0.11 | 84±45 | 68±55 | <0.0001 |
| UAC (mg/l) | 15 (84) | 1689 (2200) | <0.0001 | 21 (177) | 44 (916) | 0.17 | 19 (174) | 189 (576) | 0.02 | 19 (157) | 188 (910) | <0.0001 |
| Total cholesterol* (mmol/l) | 5.59±1.46 | 6.31±1.38 | 0.01 | 5.68±1.51 | 5.92±1.07 | 0.28 | 5.68±1.47 | 6.22±1.43 | 0.19 | 5.66±1.42 | 5.77±1.67 | 0.97 |
| Triglycerides* (mmol/l) | 1.13(0.82) | 1.49 (0.99) | 0.07 | 1.14 (0.78) | 1.40 (0.95) | 0.81 | 1.13 (0.68) | 2.45 (2.25) | 0.07 | 1.12 (0.63) | 1.56 (1.55) | 0.004 |
| Tobacco smoking^†^ (%) | 42.5 | 40.0 | 0.72 | 41.6 | 45.8 | 0.57 | 41.5 | 52.0 | 0.30 | 40.4 | 57.8 | 0.005 |
| Antihypertensive drugs (%) | 48.9 | 85.5 | <0.0001 | 51.2 | 69.4 | 0.01 | 51.2 | 80.8 | 0.003 | 50.6 | 72.2 | 0.0004 |
| ACE-I or ARB drugs (%) | 40.3 | 69.1 | <0.0001 | 41.8 | 49.0 | 0.33 | 41.8 | 50.0 | 0.41 | 41.5 | 52.8 | 0.06 |
| Lipid lowering drugs (%) | 8.4 | 12.7 | 0.28 | 8.1 | 16.3 | 0.05 | 8.4 | 11.5 | 0.57 | 8.5 | 11.1 | 0.46 |
| Diabetic nephropathy stages (%) | 54/24/18/4 | 5/0/31/64 | <0.0001 | 49/21/17/13 | 33/20/27/20 | 0.08 | 49/21/17/13 | 27/8/46/19 | 0.0006 | 49/22/17/12 | 28/11/26/35 | <0.0001 |
| Diabetic retinopathy stages (%) | 0/34/18/48 | 0/7/16/77 | <0.0001 | 0/32/17/51 | 0/14/14/72 | 0.01 | 0/32/17/51 | 0/4/27/69 | 0.009 | 0/32/17/51 | 0/13/18/69 | 0.002 |
| Peripheral sensory neuropathy (%) | 45.9 | 72.6 | 0.0002 | 46.5 | 75.0 | 0.0002 | 46.9 | 79.2 | 0.002 | 45.6 | 78.3 | <0.0001 |
| Peripheral artery disease (%) | 12.0 | 23.5 | 0.02 | 11.5 | 46.5 | <0.0001 | 12.6 | 39.1 | 0.0002 | 11.3 | 38.2 | <0.0001 |

Results expressed as means ± SD, except urinary albumin cpncentration (UAC) and triglycerides expressed as median and (interquartile range). Statistics of quantitative parameters are ANOVA performed. If the normality of the distribution was rejected by the Shapiro-Wilk W test, data was log-transformed, or Wilcoxon test was used (UAC, and triglycerides). BP, blood pressure. eGFR, estimated glomerular filtration rate computed by Modification of Diet in Renal Disease formula (MDRD) formula. ESRD, end-stage renal disease requiring haemodialysis or kidney transplantation. All-cause mortality, death during follow-up as result of cardiovascular diseases or any other causes. UAE: Urinary Albumin Excretion. *Data available only in the GENEDIAB cohort: n=438 for total cholesterol and n=129 for triglycerides. ^†^Current or past history of tobacco smoking. Antihypertensive drugs: all antihypertensive medication classes included. ACE-I: Angiotensin Converting Enzyme Inhibitor. ARB: Angiotensin Receptor Blocker. Diabetic nephropathy stages: absent, incipient, established, advanced. Diabetic retinopathy stages: absent, non-Proliferative, pre-Proliferative, proliferative. p<0.05 was significant.

**Additional file 1 - Table S4. Covariates associated with diabetic nephropathy at the end of follow-up – Stepwise Multivariable Regression Analysis**

|  | Cumulated R^2^ | ß Coefficient | p |
| --- | --- | --- | --- |
| Antihypertensive drugs | 0.052 | 0.76 | **<0.0001** |
| Diabetic retinopathy (stage 4 vs 2-3) | 0.076 | 0.46 | **<0.0001** |
| Age | 0.091 | -1.67 | **<0.0001** |
| History of LEA | 0.107 | 0.55 | **<0.0001** |
| Systolic blood pressure | 0.118 | 2.56 | **<0.0001** |
| BMI | 0.122 | -1.50 | **0.0007** |
| Lipid lowering drugs | 0.124 | 0.24 | **0.03** |
| Duration of diabetes | - | -0.47 | 0.05 |
| HbA1c | - | 0.41 | 0.29 |
| Sex (male) | - | 0.05 | 0.35 |
| Tobacco smoking | - | 0.05 | 0.48 |
| Cohort: GENEDIAB | - | 0.05 | 0.52 |
| Diastolic blood pressure | - | 0.22 | 0.70 |
| ACE-I or ARB drugs | - | -0.02 | 0.84 |

Cross-sectional analysis using for each participant the most recent set of data available at the end of follow-up. Diabetic nephropathy was coded as an ordinal polytomic covariate: absence (1), incipient (2), established (3) and advanced nephropathy (4), and ESRD (5). Cumulated R^2^ expresses the percentage of the variation of the dependent variable explained by the stepwise inclusion of independent covariates in the model (1=100%). Statistical analyses test the probability for the independent variable effect (ß Coefficient) to be different from zero. Quantitative parameters were log-transformed for the analysis. Diabetic retinopathy was coded as an ordinal polytomic covariate: non-Proliferative (2), pre-Proliferative (3), proliferative (4). Tobacco smoking: current or past history. Antihypertensive drugs: all antihypertensive medication classes included. ACE-I: Angiotensin Converting Enzyme Inhibitor. ARB: Angiotensin 2 Receptor Blocker.

**Additional file 1 - Table S5. Covariates associated with myocardial infarction at the end of follow-up – Stepwise Multivariable Regression Analysis**

|  | Cumulated R^2^ | ß Coefficient | p |
| --- | --- | --- | --- |
| History of LEA | 0.090 | 0.73 | **<0.0001** |
| Age | 0.132 | 1.55 | **<0.0001** |
| Lipid lowering drugs | 0.154 | 0.54 | **0.0004** |
| Antihypertensive drugs | 0.175 | 0.56 | **0.0007** |
| eGFR | 0.183 | -0.61 | **0.03** |
| UAC | - | 0.12 | 0.14 |
| Diabetic retinopathy (stage 4 vs 2-3) | - | 0.19 | 0.17 |
| Tobacco smoking | - | 0.19 | 0.20 |
| Duration of diabetes | - | 0.78 | 0.20 |
| BMI | - | -0.80 | 0.28 |
| HbA1c | - | 0.76 | 0.34 |
| Cohort: GENEDIAB | - | 0.14 | 0.36 |
| Sex (male) | - | 0.07 | 0.63 |
| ACE-I or ARB drugs | - | -0.06 | 0.75 |
| Systolic blood pressure | - | 0.32 | 0.79 |
| Diastolic blood pressure | - | -0.12 | 0.95 |

Cross-sectional analysis using for each participant the most recent set of data available at the end of follow-up. Cumulated R^2^ expresses the percentage of the variation of the dependent variable explained by the stepwise inclusion of independent covariates in the model (1=100%). Statistical analyses test the probability for the independent variable effect (ß Coefficient) to be different from zero. Quantitative parameters were log-transformed for the analysis. eGFR: estimated glomerular filtration rate. UAC: urinary albumin concentration. Diabetic retinopathy was coded as an ordinal polytomic covariate: non-Proliferative (2), pre-Proliferative (3), proliferative (4). Tobacco smoking: current or past history. Antihypertensive drugs: all antihypertensive medication classes included. ACE-I: Angiotensin Converting Enzyme Inhibitor. ARB: Angiotensin 2 Receptor Blocker.

**Additional file 1 - Table S6. Covariates associated with stroke at the end of follow-up – Stepwise Multivariable Regression Analysis**

|  | Cumulated R^2^ | | ß Coefficient | p |
| --- | --- | --- | --- | --- |
| Systolic blood pressure | 0.051 | | 2.42 | **<0.0001** |
| Duration of diabetes | 0.091 | | 1.88 | **0.0001** |
| UAC | 0.129 | | 0.28 | **0.0002** |
| History of LEA | 0.140 | | 0.37 | **0.04** |
| Tobacco smoking | - | | 0.16 | 0.16 |
| HbA1c | - | | 1.31 | 0.19 |
| ACE-I or ARB drugs | - | | 0.12 | 0.23 |
| Sex (male) | - | | 0.16 | 0.28 |
| Diabetic retinopathy (stage 4 vs 2-3) | | - | 0.43 | 0.30 |
| Age | - | | 1.04 | 0.35 |
| Cohort: GENEDIAB | - | | 0.15 | 0.38 |
| eGFR | - | | 0.21 | 0.46 |
| Lipid lowering drugs | - | | 0.10 | 0.67 |
| Antihypertensive drugs | - | | 0.09 | 0.75 |
| BMI | - | | -0.19 | 0.84 |
| Diastolic blood pressure | - | | 0.16 | 0.92 |

Cross-sectional analysis using for each participant the most recent set of data available at the end of follow-up. Cumulated R^2^ expresses the percentage of the variation of the dependent variable explained by the stepwise inclusion of independent covariates in the model (1=100%). Statistical analyses test the probability for the independent variable effect (ß Coefficient) to be different from zero. Quantitative parameters were log-transformed for the analysis. eGFR: estimated glomerular filtration rate. UAC: urinary albumin concentration. Diabetic retinopathy was coded as an ordinal polytomic covariate: non-Proliferative (2), pre-Proliferative (3), proliferative (4). Tobacco smoking: current or past history. Antihypertensive drugs: all antihypertensive medication classes included. ACE-I: Angiotensin Converting Enzyme Inhibitor. ARB: Angiotensin 2 Receptor Blocker.

**Additional file 1 - Table S7. Covariates associated with all-cause mortality – Stepwise Multivariable Regression Analysis**

|  | Cumulated R^2^ | ß Coefficient | p |
| --- | --- | --- | --- |
| History of LEA | 0.141 | 0.63 | **<0.0001** |
| Diabetic Nephropathy (stages 3-4-5 vs 1-2) | 0.213 | 0.94 | **<0.0000** |
| Myocardial infarction | 0.249 | 0.73 | **0.0001** |
| HbA1c | 0.140 | 3.58 | **0.0001** |
| Tobacco smoking | 0.303 | 0.45 | **0.002** |
| Cohort: GENEDIAB | 0.322 | 0.63 | **0.004** |
| BMI | 0.342 | -3.44 | **0.003** |
| Age | 0.352 | 2.04 | **0.03** |
| Diabetic Nephropathy (stage 5 vs 3-4) | 0.362 | 0.59 | **0.03** |
| Diabetic retinopathy (stage 4 vs 2-3) | - | 0.36 | 0.13 |
| Stroke | - | 0.45 | 0.18 |
| ACE-I or ARB drugs | - | -0.18 | 0.19 |
| Duration of diabetes | - | 0.87 | 0.22 |
| Systolic blood pressure | - | 0.92 | 0.30 |
| Sex (male) | - | 0.24 | 0.31 |
| Lipid lowering drugs | - | -0.21 | 0.46 |
| Diastolic blood pressure | - | -0.41 | 0.78 |
| Antihypertensive drugs | - | -0.03 | 0.92 |

Cross-sectional analysis using for each participant the most recent set of data available at the end of follow-up. Cumulated R^2^ expresses the percentage of the variation of the dependent variable explained by the stepwise inclusion of independent covariates in the model (1=100%). Statistical analyses test the probability for the independent variable effect (ß Coefficient) to be different from zero. Quantitative parameters were log-transformed for the analysis. Diabetic nephropathy was coded as an ordinal polytomic covariate: absence (1), incipient (2), established (3) and advanced nephropathy (4), and ESRD (5). Diabetic retinopathy was coded as an ordinal polytomic covariate: non-Proliferative (2), pre-Proliferative (3), proliferative (4). Tobacco smoking: current or past history. Antihypertensive drugs: all antihypertensive medication classes included. ACE-I: Angiotensin Converting Enzyme Inhibitor. ARB: Angiotensin 2 Receptor Blocker.
